# CD44 and SLC1A2 are commonly regulated but do not form a fusion transcript in ER+ breast cancer

**DOI:** 10.1007/s11010-025-05308-w

**Published:** 2025-05-23

**Authors:** Francesca Bonechi, Marina Bacci, Nicla Lorito, Alfredo Smiriglia, Edoardo Pagliantini, Matteo Benelli, Icro Meattini, Andrea Morandi

**Affiliations:** 1 Department of Experimental and Clinical Biomedical Sciences, University of Florence, Viale Morgagni 50, 50134 Florence, Italy; 2 Radiation Oncology Unit, Oncology Department, Azienda Ospedaliero Universitaria Careggi, Largo Brambilla 3, 50134 Florence, Italy

**Keywords:** Breast cancer, Endocrine therapy, Metabolic reprogramming

## Abstract

Endocrine therapy (ET) is essential for managing ER+ HER2− breast cancer; however, resistance remains a significant clinical challenge. This study investigated whether CD44-SLC1A2 gene fusions, reported in gastrointestinal malignancies, contribute to ET resistance mechanisms in breast cancer. Although no CD44-SLC1A2 fusions were detected, high expression of CD44 and SLC1A2 was associated with poor survival outcomes and identified a therapy-resistant subpopulation sustained by aspartate and glutamate metabolism, highlighting potential metabolic vulnerabilities for future therapeutic intervention.

## Introduction

Interfering with estrogen receptor (ER) activity using endocrine therapy (ET) is the cornerstone of treatment for early and advanced ER+ HER2− breast cancers. Despite its clinical efficacy, overcoming both *de novo* and acquired therapy resistance remains an unmet clinical need [1]. While some resistance cases can be attributed to genetic alterations, such as CYP19A1 amplification or ESR1 mutations [2, 3], a large proportion stems from non-genetic mechanisms. Among these, metabolic reprogramming significantly contributes to therapy resistance [4–7].

We have previously reported that ET-resistant breast cancers exhibit enhanced expression of the glutamate and aspartate transporter SLC1A2, which supports the metabolic demands sustaining tumor aggressiveness [5]. Notably, increased intracellular amino acid levels—particularly aspartate—have been shown to play a crucial role in promoting tumor progression [8, 9]. Furthermore, ER+ breast cancer cells with high levels of CD44 show increased phenotypic plasticity and resistance under ET pressure [10, 11]. The apparent independence of these features in ET-resistant breast cancers prompted us to investigate potential interactions between CD44 and SLC1A2, hypothesizing that they might share regulatory mechanisms. One possible mode of interaction is through gene fusion events, which are increasingly recognized as drivers of oncogenic behavior. Several observations support this hypothesis: (a) CD44 and SLC1A2 genes are located in close proximity on chromosome 11 (11p13); (b) CD44-SLC1A2 gene fusions have been detected in gastric and colorectal cancers [12, 13]; (c) these gene fusions can occur independently of 11p13 amplification [13]; (d) the CD44-SLC1A2 gene fusion produces a truncated yet functional SLC1A2 protein that maximizes the uptake of aspartate and glutamate [13]; (e) silencing SLC1A2 in CD44-SLC1A2 fusion-carrying models reduces the intracellular levels of aspartate and glutamate, thereby impairing aggressive features and sensitizing gastric cancers to chemotherapy [13]. Chromosomal proximity is a key factor in gene fusion formation, as genes located near each other are more likely to undergo aberrant recombination, such as those observed in the CD44-SLC1A2 fusions. These points collectively warrant further investigation into the potential regulatory mechanisms involving CD44 and SLC1A2 in ET-resistant breast cancers. Moreover, it is crucial to explore whether CD44-SLC1A2 fusions are specific to gastrointestinal (GI) or also occur in breast cancers.

## Material and methods

### Cell lines and reagents

Wild-type (WT) MCF7 cells were obtained from ATCC and maintained in phenol red–free RPMI1640 supplemented with 10% FBS (Euroclone), 2 mmol/L glutaMAX (Gibco), and 1 nmol/L 17-β estradiol (E2, Sigma). Long-term estrogen deprived (LTED) MCF7 cells were cultured in steroid-depleted phenol red–free RPMI1640 medium containing 10% dextran charcoal-stripped (DCC) FBS (Hyclone) and 2 mmol/L glutaMAX (DCC medium). Cells were short tandem repeat tested, amplified, stocked, routinely subjected to mycoplasma testing, and once thawed were kept in culture for a maximum of 3 weeks.

### Western blot analysis

Western blot analyses were conducted as described previously [14]. Briefly, cells were lysed in RIPA buffer and 20–50 μg of total proteins were loaded on precast SDS-PAGE gels (Bio-Rad). The following antibodies were used: CD44 (Cell Signaling, 156-3 C11), SLC1A2 (Santa Cruz Biotechnology, sc-365634), HSP90 (Santa Cruz Biotechnology, sc-69703). HSP90 was used as loading control and the quantification of each band was reported as normalized to the loading control.

### Quantitative real-time PCR (qRT-PCR) analysis

Total RNA was extracted using RNeasy Kit (Qiagen) and cDNA synthesis was performed using Reverse Transcription Kit (Applied Biosystems). qRT-PCR was conducted as described previously [15] using TaqMan assays. The following probes were used: CD44 (Thermo Fisher Scientific, Hs01075864_m1) and SLC1A2 (Thermo Fisher Scientific, Hs01102423_m1). Additionally, a custom-designed assay was used to detect CD44-SLC1A2 with the primers specified in [13]. Data were normalized on TBP (TATA-Box Binding Protein, Thermo Fisher Scientific, Hs00427620_m1). The relative quantity was determined using ΔΔCt by the CFX Maestro software (Bio-Rad).

### Transient siRNA transfection

Cells were transfected with 30 nmol/L siRNA targeting SLC1A2 (Sigma, Hs01_00109453), CD44 (Sigma, SASI_Hs02_00302917) or negative control (siCTR, Sigma, SIC001) using RNAiMAX Reagent (Thermo Fisher Scientific #13778-150) and Opti-MEM (GIBCO) accordingly to manufacturer’s instructions and as previously reported [16]. The functional analyses were performed 72 h after transfection.

### Immunofluorescence

Glass coverslip-plated cells were fixed with 4% formaldehyde for 1 h, then permeabilized with 0.1% Triton X-100 in PBS before incubation with primary antibodies overnight at 4 °C. Secondary antibodies conjugated with AlexaFluor488 or 633 were incubated for 1 h at room temperature. All fluorescence samples were examined at room temperature using a microscope (TCS SP8; Leica) with lasers exciting at 488, 543, and 633 nm.

### *In silico* analysis

Multivariate analysis was conducted using the finalfit (v. 1.0.8) software in R. A Cox proportional hazards regression model was employed, with overall survival as the dependent variable. The independent variables included in the model were: classification based on the expression levels of the CD44 and SLC1A2 genes, patient age, MKI67 gene expression, PAM50 molecular subtype, tumor stage, lymph node involvement, and a Relapse/Dead variable. This variable classified patients into two groups: early relapse or death events within 5 years versus all others.

### Colony formation assay

To evaluate the clonogenicity of breast cancer cells, 0.5 × 10^3^ cells were seeded per well in a 6-well plate after transfection with 30 nmol/L siCTR, siCD44, or siSLC1A2. 15 days post-seeding, the colonies were fixed with 4% formaldehyde and stained with crystal violet staining solution. Air-dried plates were scanned, and images analyzed using Image J.

### Statistical analysis

Statistical analysis was conducted using GraphPad Prism Software. Comparisons between 2 groups were made using the two-tailed, unpaired Student’s t-test. Comparisons between multiple groups were made using one-way analysis of variance (ANOVA) and Tukey post testing analyses with a confidence interval of 95% used for individual comparisons. Statistical significance was defined when *P* < 0.05. *P*-values are indicated in Figure Legends.

## Results and discussion

**Fig. 1:**
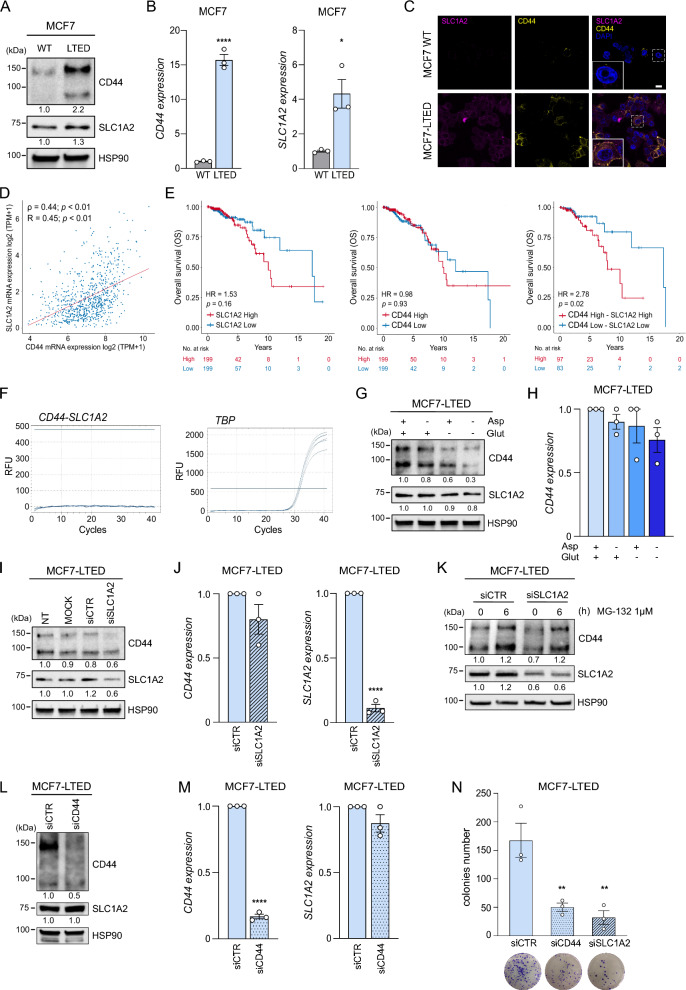
CD44 and SLC1A2 are co-regulated without fusion in ER+ breast cancer. **A** Total protein lysate from MCF7 parental cells (WT) and the corresponding LTED derivatives were subjected to western blot analysis using the reported antibodies. **B** Total RNA was extracted from LTED-derived and parental MCF7 cells and analyzed by qRT-PCR using the indicated probes. Data were normalized on TBP and reported as fold change over the comparator set at 1. **C** Parental and MCF7-LTED cells were subjected to CD44 and SLC1A2 content immunofluorescence analysis by confocal microscopy. Representative pictures of CD44 and SLC1A2 are shown with a higher magnification represented in the highlighted inset (magenta: SLC1A2; yellow: CD44; blue: DAPI, nuclei; scale bar, 10 μm). **D** Correlation analysis between the expression levels of CD44 and SLC1A2 in a cohort of 794 ER+ breast cancer patients from the TCGA database. The analysis shows a positive correlation between the expression levels of CD44 and SLC1A2. **E** Kaplan–Meier analysis of OS in the TCGA cohort of ER+ breast cancer patients dichotomized based on higher and lower mRNA expression of SLC1A2 and/or CD44 using the upper and lower quartile. HR and log-rank Mantel–Cox *P*-values are shown. **F** Total RNA was extracted from MCF7-LTED cells and analyzed by qRT-PCR using a custom-designed TaqMan probe assay to detect the SLC1A2-CD44 fusion mRNA. The qRT-PCR amplification plots show no SLC1A2-CD44 fusion transcript. **G**, **H** MCF7-LTED cells were cultured in the presence of all the amino acids or in the absence of aspartate and/or glutamate for 48 h. **G** Total protein lysates were subjected to western blot analysis using the indicated antibodies. **H** Total RNA extracted was analyzed by qRT-PCR for CD44 expression. Data were normalized on TBP and reported as fold change over the comparator set at 1. **I** MCF7-LTED cells were transfected with non-Targeting siRNA (siCTR) or siRNA targeting SLC1A2 (siSLC1A2) for 72 h and total protein lysate was subjected to western blot analysis using the indicated antibodies. **J** MCF7-LTED cells transfected with siCTR or siSLC1A2 for  72 h were subjected to RNA extraction to analyze SLC1A2 and CD44 expression by qRT-PCR. Data were normalized on TBP and reported as fold change over the comparator set at 1. **K** MCF7-LTED cells transfected with siCTR or siSLC1A2 were treated with the proteasome inhibitor MG-132 (1 μM) for 6 h and total protein lysate was subjected to western blot analysis using the indicated antibodies. **L** MCF7-LTED cells were transfected with siCTR or siRNA targeting CD44 (siCD44) and total protein lysate was subjected to western blot analysis using the indicated antibodies. **M** MCF7-LTED cells transfected with siCTR or siCD44 for 72 h were subjected to RNA extraction to analyze SLC1A2 and CD44 expression by qRT-PCR. Data were normalized on TBP and reported as fold change over the comparator set at 1. **N** MCF7-LTED cells transfected with siCTR, siCD44, or siSLC1A2 were subjected to colony formation assay and colonies number was quantified using Image J. Data are mean ± SEM. *P < 0.05; **P < 0.01; ****P < 0.0001

Long-term estrogen deprived (LTED) cells represent a well-established model for studying ET resistance and have been comprehensively characterized in terms of their molecular and metabolic properties [4–6]. To test our hypothesis, we subjected the ER+ MCF7 breast cancer cells and their LTED derivatives to western blotting (Fig. 1A), quantitative real-time PCR (qRT-PCR, Fig. 1B), and confocal microscopy (Fig. 1C). SLC1A2 and CD44 expression levels were significantly elevated in LTED cells at both the mRNA and protein levels compared to their parental counterparts. However, no evident colocalization was observed from the confocal analysis (Fig. 1C). To further explore this, we interrogated publicly available transcriptomic data from breast cancer patients in the TCGA database. The analysis revealed a positive correlation between SLC1A2 and CD44 expression levels (*R* = 0.45, *P* < 0.01, Fig. 1D). High expression of either gene was independently associated with reduced overall survival (OS) in ER+ breast cancer patients, although the correlation did not reach statistical significance (SLC1A2 HR = 1.53, *P* = 0.16, CD44 HR = 0.98, *P* = 0.93; Fig. 1E). However, tumors with concomitantly high expression of both genes, defined by upper and lower quartiles, showed a significant association with reduced OS (HR = 2.78, *P* = 0.02, Fig. 1E), which remained significant after multivariate analysis (Table 1), highlighting potential clinical relevance.

**Table 1:** Multivariate analysis

Variable	All or mean (% or SD)	HR univariate (CI; *P*-value)	HR multivariate (CI; * P*-value)
CD44-SLC1A2			
High–high	97 (53.9)	–	–
Low–low	83 (46.1)	0.36 (0.15–0.88, *P* = 0.026)	0.23 (0.05–0.99, *P* = 0.048)
Age			
Mean (SD)	59.4 (13.9)	1.06 (1.03–1.09, *P* < 0.001)	1.08 (1.02–1.13, *P* = 0.006)
Relapse/dead			
≤ 5 years	13 (18.1)	–	–
Relapse/dead/alive			
> 5 years	59 (81.9)	0.29 (0.11–0.78, *P* = 0.014)	0.25 (0.05–1.26, *P* = 0.093)
MKI67			
Mean (SD)	4.2 (1.2)	1.06 (0.75–1.49, *P* = 0.749)	0.94 (0.60–1.47, *P* = 0.781)
Subtype			
Luminal A	110 (69.2)	–	–
HER2	8 (5.0)	1.48 (0.30–7.37, P = 0.632)	17.05 (1.43–203.45, *P* = 0.025)
Luminal B	41 (25.8)	1.50 (0.57–3.95, *P* = 0.410)	2.40 (0.64–9.03, *P* = 0.194)
Stage			
Stage I	40 (22.7)	–	–
Stage II	94 (53.4)	1.19 (0.41–3.43, *P* = 0.744)	0.25 (0.05–1.25, *P* = 0.091)
Stage III	42 (23.9)	1.23 (0.36–4.28, *P* = 0.740)	0.20 (0.02–1.63, *P* = 0.132)
Lymphnodes			
N+	86 (49.4)	–	–
N0	88 (50.6)	0.62 (0.26–1.45, *P* = 0.271)	0.19 (0.04–0.99, *P* = 0.049)

Number in data frame = 180, number in model = 60, missing = 120, number of events = 18, concordance = 0.859 (SE = 0.046), *R*^2^ = 0.392 (max possible = 0.846), Likelihood ratio test = 29.813 (*df* = 9, *P* = 0.000)

To determine whether CD44 and SLC1A2 expression arises from independent transcription or a fusion construct, as reported in GI cancers, we employed a dual experimental approach. First, we performed qRT-PCR using a custom-designed TaqMan probe to detect CD44-SLC1A2 fusion mRNA in MCF7-LTED cells. No amplification signal was observed, indicating the absence of the fusion transcript (Fig. 1F) and suggesting independent transcription. Consistently, analysis of TCGA, FusionGDB2.0, Mitelman, and COSMIC databases identified five CD44-SLC1A2 fusions in GI cancers (out of 1271 GI patients), but none in breast cancers (1618 patients analyzed, data not shown).

Although the CD44-SLC1A2 fusion variant was absent in MCF7-LTED cells, we investigated potential interactions between SLC1A2 and CD44. Specifically, we examined whether SLC1A2 function influences CD44 expression and vice versa. Interestingly, aspartate/glutamate deprivation led to reduced CD44 protein levels (Fig. 1G) without significantly altering CD44 mRNA levels (Fig. 1H). Similarly, SLC1A2 knockdown reduced CD44 protein expression (Fig. 1I), while leaving CD44 mRNA levels unaffected (Fig. 1J), suggesting a post-transcriptional regulation mechanism. Indeed, MCF7-LTED cells subjected to SLC1A2 silencing and treated with the proteasome inhibitor MG-132 showed unchanged CD44 protein levels (Fig. 1K), indicating that SLC1A2 regulates CD44 protein stability or turnover, rather than its transcription.

To evaluate whether this regulatory mechanism is bidirectional, we silenced CD44 and assessed SLC1A2 expression. In contrast to SLC1A2 silencing, CD44 knockdown did not affect SLC1A2 expression at either protein (Fig. 1L) or mRNA levels (Fig. 1M). However, silencing either CD44 or SLC1A2 reduced the clonogenic potential of MCF7-LTED cells, a trait associated with aggressiveness and resistance (Fig. 1N). Collectively, these findings suggest that the contribution of SLC1A2 to endocrine resistance is influenced by CD44 expression, whereas CD44 acts independently of SLC1A2. This supports the hypothesis that concomitant higher expression of SLC1A2 and CD44 marks a potentially aggressive subpopulation within ER+ breast tumors, distinct from those with high levels of only one of the two genes. This subpopulation may contribute to resistance and disease progression, even in the absence of a CD44-SLC1A2 fusion. Additionally, aspartate and glutamate appear critical for maintaining CD44 expression, reinforcing their role in supporting aggressive, stem-like cancer features.

Further studies are needed to validate these findings in larger cohorts of ET-treated breast cancer patients and to investigate whether CD44-SLC1A2 fusions may emerge in specific subgroups. Expanding this analysis to other breast cancer subtypes may uncover novel mechanisms and biomarkers, ultimately improving patient prognosis and guiding therapeutic strategies.

## Data Availability

Data will be made available from the corresponding author upon request.

## Abbreviations

ER: Estrogen receptor
ET: Endocrine therapy
HER2: Human epidermal growth factor receptor 2
LTED: Long-term estrogen deprived
qRT-PCR: Quantitative real-time PCR
SLC: Solute carrier
